# Proper Method of Percussing the Chest

**Published:** 1894-03

**Authors:** Thomas J. Mays

**Affiliations:** Philadelphia; Professor of Diseases of the Chest in the Philadelphia Polyclinic


### THE PROPER METHOD OF PERCUSSING THE CHEST.

By THOMAS J. MAYS, A. M., M. D.,
Professor of Diseases of the Chest in the Philadelphia Polyclinic.

Percussion is the art of eliciting sounds from the body by gently tapping or striking it. From the nature of these sounds we are enabled to determine many of the physical conditions of the thoracic organs in health and in disease. The first practical question that arises in connection with this subject is as to the best method of percussing the chest.

Always become accustomed to use the fingers as percussion instruments. These are preferable because they are never absent, are readily adjusted to the intercostal spaces, and fossæ of the chest, and are endowed with a delicate touch with which we are able to perceive the hardness, softness or resistance which may be present.

Always recognize that while percussing the relative position between your ear and the patient's chest must be disturbed as little as possible; for percussion is a process by which you compare sounds obtained from opposite sides of the chest, and in order to be able to make an intelligent comparison like conditions must be maintained. Hence stand directly in front of or behind the patient, as the case may be, and do not walk from one side of the patient to the other as you percuss around his body.

For a pleximeter, lay one finger of the left hand, preferably the second, firmly against the chest wall, and in such a direction that the distal end points outward, and the central end toward the sternum if you percuss in front, and the latter end toward the spine if you percuss on the back. This will make it easy for you to maintain a central position before or behind your patient, and will also preserve that essential relative uniform condition of both sides of the chest, which would be impossible if you laid your finger on the chest, pointing at random, in any direction. As a plexor use the second finger of the right hand. This should have its nail well trimmed, and should be bent at a right angle at the second joint. Deliver your blows gently, and always perpendicularly to the chest wall, and never strike from the elbow, but only from the wrist or knuckle.

Do not percuss over a rib on one side and over an intercostal space on the other, nor percuss down one side of the chest, then down the other side. For, as already stated, percussion is a comparing process, and if this is not observed, it will be found that by the time the bottom of one side is reached the exact pitch of the sounds which were heard at the apex will be more or less dimmed or actually forgotten, and there will be nothing reliable with which to compare the percussion sounds of the opposite side. Therefore, always percuss over symmetrical areas; first over one supra-clavicular region, then over the other; next, one infra-clavicular region, then the other, and so on down to the base of the chest in front, and then behind, remembering, however, that the percussion pitch is slightly higher over the right than over the left apex.

Never omit immediate clavicular percussion, for you will often be able to detect delicate differences of sound here which would otherwise be lost. Do not stand your patient against the wall, nor let him lean against any object, nor allow the arms to be folded, but direct that these shall hang loosely by the patient's side, with a slight forward inclination. If your patient is confined to bed, endeavor, if not too seriously ill, to get him in the sitting position. If this is impossible, change his position in bed in such a way that you will be able to keep the proper relation to the two sides of his body and make a satisfactory examination.—*The C. and C. Record*.

